# Parturition en présentation transverse et modalités d'accouchement: la césarienne versus la manœuvre de version interne et grande extraction du siège (MVIGES); à propos de 162 accouchements à la maternité Jason Sendwe de Lubumbashi, RD du Congo

**DOI:** 10.11604/pamj.2014.19.300.3120

**Published:** 2014-11-18

**Authors:** Julien Kimbala, Olivier Mukuku, Xavier Kinenkinda, Justin Kizonde

**Affiliations:** 1 Département de Gynécologie-Obstétrique, Cliniques Universitaires de Lubumbashi, Faculté de Médecine, Université de Lubumbashi, République Démocratique du Congo

**Keywords:** Présentation transverse, césarienne, version par manœuvre interne, grande extraction du siège, morbi-mortalité fœto-maternelle, transverse presentation, caesarean section, internal version maneuver, large breech extraction, maternal-fetal morbidity and mortality

## Abstract

**Introduction:**

Le but de ce travail est de déterminer la fréquence des parturitions en présentation transverse dans notre milieu, décrire les particularismes sociodémographiques et l'environnement obstétrical y afférents et évaluer la morbi-mortalité maternelle et périnatale en rapport avec les différentes modalités d'accouchement.

**Méthodes:**

Il s'agit d'une étude rétrospective portant sur 162 accouchements consécutifs avec fœtus en présentation transverse, d’âge gestationnel supérieur à 27 semaines aménorrhées, enregistrés au cours de la période allant de Janvier 2002 à Décembre 2006 sur un total de 53679 accouchements à la maternité de l'Hôpital Général de Référence Jason Sendwe de Lubumbashi (RDCongo). Les 162 accouchées et leurs nouveau-nés ont été repartis en 3 groupes selon les modalités d'accouchement: césarienne, voie basse (VB) après MVIGES sur J2 et VB après MVIGES sur singleton. Les paramètres sociodémographiques, l'environnement obstétrical ainsi que la morbi-mortalité fœto-maternelle ont été analysés. Sauf indication contraire, les comparaisons statistiques entre les groupes ont été effectuées par rapport au groupe d'accouchées par césarienne.

**Résultats:**

La parturition en présentation transverse concerne une grossesse sur 333. L'accouchement par VB après MVIGES a été le principal mode de terminaison (60,5%) représentant 95% de succès de cette manœuvre. La MVIGES a concerné 50,86% des grossesses singletons dont un peu plus de la moitié avec fœtus vivants et 84,78% des deuxièmes jumeaux dont 91,7% vivants. L’âge moyen et la parité moyenne des accouchées sont comparables dans les trois groupes (p = 0,17 et p = 0,84). 61% des grossesses terminées par voie haute étaient à terme contre 42% pour celles de la VB (p = 0,17). Le poids de naissance moyen des nouveau-nés par voie haute (2780g) était statiquement comparable à celui des singletons nés par VB (2380g), il en est de même de la proportion des nouveau-nés avec un poids supérieur à 2499g (p = 0,102). Le taux d’évacuées s’élève à 80% dans les parturitions singletons avec 96% des membranes rompues et une mortinatalité 3 fois plus élevée comparativement au contenu gémellaire. La morbidité maternelle est caractérisée par un taux d'infection de 9,4% parmi les césarisées (p = 0,0000) et un taux de complications traumatiques de 3,4% chez les accouchées par VB après MVIGES (p = 1,0000). Le taux de dépression néonatale à la fin de la première minute est tout aussi fort élevé chez les nouveau-nés par césarienne (66%) que ceux singletons nés par VB (60,7%, p = 0,82), les J2 nés par VB ayant présenté une moindre morbidité (31%, p = 0,0035). Au-delà de la 4ème minute, le risque de dépression néonatale n'est pas différent quelque soit la modalité d'accouchement considérée (OR = 1,75: 0,41-7,86 et OR = 0,50: 0,14-1,74). Les taux de déperdition néonatale sont de 23% pour les J2 nés par VB, de 12% et 11% pour les nouveau-nés par césarienne et les singletons nés par VB, ils ne sont pas statistiquement différents (p = 0,31). Enfin, le séjour hospitalier moyen est significativement plus long pour les accouchées par césarienne (12,97 jours) par rapport aux accouchées par VB (2,54 et 2,21 jours) (p = 0,000) alors que celui des nouveau-nés n'est pas statistiquement différent (3,25; 5,2 et 2,6 jours; p = 0,09).

**Conclusion:**

La parturition en présentation transverse n'est pas un événement rare dans notre milieu, elle est caractérisée par un environnement obstétrical précaire et la MVIGES (pratiquée dans le strict respect des conditions de sa réalisation et des contre-indications de la VB) est une opération faisable, non délétère et non fœticide et qu'elle constitue de ce fait une alternative véritablement heureuse à la césarienne.

## Introduction

Les parturitions en présentation transverse représentent un cas sur 200 à 500 [1–3]. A première vue, cette incidence pourrait paraître négligeable mais si l'on se place dans le contexte des populations à forte fécondité et éthnoculturellement pronatalistes cela constitue à coût sûr un réel problème de santé dans la pratique obstétricale quotidienne. L'accouchement en transverse requiert le recours à la césarienne car sans elle, l’évolution spontanée conduit à des dégâts incalculables: mort fœtale, rupture utérine, choc hypovolémique... aboutissant bien souvent au décès maternel [1, 2, 4]. La voie basse (VB) n'est envisageable que pour le deuxième jumeau (J2) en transverse grâce à la version par manœuvre interne (VMI) indiquée après expulsion du premier bien entendu ou alors concernant des tout-petits fœtus ou macérés pouvant eux être expulsés pliés en deux (conduplicato corpore) [1, 2, 4]. Considérée à tors ou à raison comme délétère et fœticide par beaucoup d'auteurs [4–9], la manœuvre de version interne et grande extraction du siège (MVIGES) est devenue rare dans les pays développés car supplantée par la césarienne, jugée plus sécurisante pour le couple Mère-Enfant [10–13]. En effet, la recrudescence de la césarienne dans les pays développés s'est accompagnée d'un bénéfice proportionnel pour le couple Mère-Enfant. Aujourd'hui, avec des taux de césariennes avoisinant un accouchement sur quatre, la mortalité maternelle y afférente est inférieure à 0,003 et une mortalité périnatale presque nulle [10, 14–17]. Pourtant sur le même « vaisseau spatial » Terre, les pays non développés sont restés à l’écart de tous ces exploits technologiques, les différentes statistiques rapportées à travers les pays d'Afrique sub-saharienne montrent que l'opération césarienne reste dangereuse pour le couple Mère-Enfant dans nos conditions de travail avec des taux de mortalité maternelle et périnatale 300 fois supérieurs à ceux observés dans les pays nantis [18–22]. D'où la nécessité de réfléchir à des solutions alternatives plus sécurisantes et adaptées aux contraintes du biotope, c'est notamment le recours, au-delà de son indication classique et indéniable sur le J2, à la MVIGES pour la terminaison des parturitions en transverse des grossesses à contenu singleton.

Suite aux énormes difficultés de réaliser dans un délai raisonnable la césarienne et vue la morbidité fœto-maternelle constatée chez les opérées dans ces conditions, le service de Gynéco-Obstétrique de l'Hôpital Général de Référence Jason Sendwe de Lubumbashi avait en l'an 2000 opté pour une pratique systématique de la MVIGES en première option pour toutes les parturientes arrivant à la maternité avec un fœtus en présentation transverse, à dilatation complète et en absence de toute contre-indication de la VB: bassin vicié, utérus cicatriciel, macrosomie..., le recours à la césarienne étant dès lors réservé aux parturientes ne remplissant pas les conditions précitées ou en cas d’échec de la MVIGES. Ni l’état fœtal (bruits du cœur fœtal présents ou absents), ni celui des membranes (intactes ou rompues) ne pouvaient conditionner le choix de la modalité d'accouchement. La présente étude a pour objectifs de déterminer la fréquence des parturitions en présentation transverse dans notre milieu, de décrire les particularismes sociodémographiques et l'environnement obstétrical y afférents et d’évaluer la morbi-mortalité maternelle et néonatale en rapport avec les différentes modalités d'accouchement.

## Méthodes

L’étude porte sur 162 accouchements consécutifs avec fœtus en présentation transverse, d’âge gestationnel supérieur à 27 semaines aménorrhées, réalisés au cours de la période allant de Janvier 2002 à Décembre 2006 (5 ans) sur un total de 53679 accouchements à la maternité de l'Hôpital Général de Référence Jason Sendwe de Lubumbashi. Les 162 accouchées et leurs nouveau-nés ont été repartis en trois groupes selon les modalités d'accouchement: le groupe I constitué par les accouchées par césarienne (n = 64), le groupe II qui comprend les accouchées par VB par MVIGES du deuxième jumeau (J2) en présentation transverse après accouchement spontané du premier jumeau (J1) (n = 39) et le groupe III formé des accouchées par VB après MVIGES chez les parturientes porteuses de grossesse monofœtale (singleton) en présentation transverse (n = 59). Le recours à la MVIGES était conditionné par une dilatation complète et l'absence de contre-indication pour l'accouchement par VB. A part les huit cas pour lesquels l'on a eu recours à narco-analgésie les autres MVIGES (91,8%) ont été réalisées sur la table d'accouchement en salle des naissances sans aucune anesthésie ni tocolyse. Notons qu'il y a eu 5 tentatives de MVIGES soldées par un échec et sanctionnées par une césarienne.

Les données en rapport avec les paramètres sociodémographiques (âge maternel, parité), l'environnement obstétrical (âge gestationnel, mode d'admission à la maternité, état fœtal, état des membranes) et les résultats post-partals et néonatals ont été codifiées, puis saisies à l'ordinateur dans son langage Access et les analyses statistiques réalisées sur le logiciel Epi Info (version 3.3.2, CDC atlanta; OMS 2010). Les moyennes sont présentées avec les écarts types (DS) et les Odds ratio (OR) avec un intervalle de confiance à 95% (IC à 95%: méthode de Cornfield). Les comparaisons statistiques entre les groupes d'accouchées ont été effectuées par rapport au groupe d'accouchées par césarienne (groupe I) sauf indication contraire. Le test Anova a été utilisé pour la comparaison des moyennes et celle des fréquences (exprimées en pourcentage) par les tests de X2 corrigé de Yates ou le test exact de Fisher lorsque recommandé. Le seuil de signification a été fixé à p < 5%. Les ajustements ont été réalisés par la méthode de régression logistique.

## Résultats

### Fréquence des parturitions en présentation transverse et modalités d'accouchement

La fréquence de parturition en présentation transverse est de 0,3% dont 71,6% des grossesses monofœtales (Tableau 1). Le taux d'accouchements par voies naturelles après la MVIGES est de 60,5%: 50,86% des grossesses singletons et 84,78% des J2.

**Tableau 1:** Fréquence des parturitions en présentation transverse et modalités d'accouchement

Présentation	Effectif	Pourcentage
**Longitudinales**	53517	99,7
601 (VH*)		
52916 (VB** )		
**Transverses**	162	0,3
59 monofætales (VB)		
57 monofætales (VH)		
07 pour J2 (VH)		
39 pour J2 (VB)		
Total	53679	100

* Voie haute

** Voie basse

### Caractéristiques sociodémographiques et environnement obstétrical (Tableau 2)

L’âge maternel s’étend de 17 à 45 ans autour d'une moyenne de 28,55 ans (DS = 5,98) dans le groupe I contre 18 à 37 ans avec une moyenne de 26,37 ans (DS = 4,93) et 17 à 41 ans avec un âge moyen de 26,98 ans (DS = 6,66) respectivement dans le groupe II et III. La parité elle, s’étend de 0 à 8 dans le groupe I autour d'une moyenne de 2.75 (DS = 1,99) et de 0 à 7 et 0 à 9 dans les groupes II et III avec des moyennes respectives de 3.03 (DS = 2,03) et 2,89 (DS = 2,49). L'analyse statistique ne montre pas de différence statistiquement significative entre les moyennes d’âge (p = 0,1763) et de parité (p = 0,84) de même que dans la comparaison des différentes tranches. L’âge gestationnel varie entre 30 et 43 SA avec un âge gestationnel moyen de 38,15 SA (DS = 2,74) dans le groupe I contre 28 et 40 SA et 28 et 44 SA pour les groupes II et III avec des moyennes respectives de 36,09 SA (DS = 3,07) et 36,7 SA (DS = 3,55). L’âge gestationnel moyen des parturientes du groupe I est statistiquement supérieur à celui de deux autres groupes (p= 0,0142). Par contre la comparaison de taux de grossesses à terme (âge gestationnel >37 SA) ne donne pas de différence statistique significative entre les trois groupes (p = 0,17 et 0,10). Concernant le mode d'admission à la maternité, la presque totalité des parturientes du groupe III (98,3%) étaient des évacuées obstétricales contre 61,3% dans le groupe I et 12,8% dans le groupe II et l'analyse statistique montre une différence hautement significative en défaveur des gestantes du groupe III et celle du groupe I (p = 0,0000). Quant à la vitalité fœtale à l'admission, les bruits cardiaques fœtaux (BCF) étaient absents dans près d'un cas sur deux (49,2%) chez les parturientes du groupe III contre respectivement 11,3% et 10,3% pour les groupes I et II et la différence est statistiquement significative en défaveur du groupe III (p = 0,0000). L’état des membranes fœtales à l'admission: elles étaient déjà rompues chez 95,2% des parturientes du groupe I et chez 96,6% de celles du groupe III (p = 0,1020) alors qu'elles ne l’étaient que dans 33,3% dans le groupe II (p = 0,0000). Le poids de naissance a varié entre 1240 et 4370 grammes dans le groupe I, 1080 et 2900 grammes dans le groupe II et entre 950 et 3600 grammes dans le groupe III. Les poids moyens de naissance sont respectivement de 2780 grammes (DS = 638,94), 1998 grammes (DS = 511,80) et 2380 grammes (DS = 686,79). Le poids moyen des nouveau-nés du groupe II est statistiquement inférieur à celui des nouveau-nés des autres groupes (p = 0,0006). Par ailleurs, les nouveau-nés ayant pesé plus de 2499 grammes représentent respectivement 63,93% et 47,46% dans les groupes I et III (p = 0,1024) contre 18,42% dans le groupe II (p = 0,000).

**Tableau 2:** Caractéristiques sociodémographiques des populations et environnement obstétrical

	Groupe I		Groupe II			Groupe III		
	n	%	N	%	p*	N	%	p*
**Age maternel**								
< 18 ans	1	1,70	0	0,00	1,0000	1	1,80	1,0000
18-35 ans	50	86,20	36	94,74	0,3062	49	85,90	0,8165
> 35 ans	7	12,10	2	5,26	0,3115	7	12,30	0,8022
	(n = 58)		(n = 38)			(n = 57)		
**Parité**								
0	6	11,70	2	6,25	0,4767	6	12,77	0,8749
1-2	23	45,10	12	37,50	0,6498	17	36,17	0,4885
3-4	11	21,60	10	31,25	0,4665	11	23,40	0,9802
≥5	11	21,60	8	25,00	0,9252	13	27,66	0,6416
	(n = 51)		(n = 32)			(n = 47)		
**Age gestationnel**								
< 32 SA	2	4,87	4	11,43	0,4050	4	8,33	0,6827
32-34 SA	0	0,00	6	17,14	0,0074	7	14,58	0,0139
35-37 SA	14	34,16	10	28,57	0,7843	17	35,42	0,9220
> 37 SA	25	60,97	15	42,86	0,1781	20	41,67	0,1080
	(n = 41)		(n = 35)			(n = 48)		
**Mode d'admission**								
Non évacuation	24	38,70	34	87,20		1	1,70	
Evacuation	38	61,30	5	12,80	0,0000	58	98,30	0,0000
	(n = 62)		(n = 39)			(n = 59)		
**Vitalité fætale**								
BCF présents	55	88,70	35	89,70		30	50,80	
BCF absents	7	11,30	4	10,30	1,0000	29	49,20	0,0000
	(n = 62)		(n = 39)			(n = 59)		
**Etat des membranes**								
Intactes	3	4,80	26	66,67		2	3,40	
Rompues	59	95,20	13	33,33	0,0000	57	96,60	1,0000
	(n = 62)		(n = 39)			(n = 59)		
**Poids de naissance**								
< 2500 grammes	22	36,10	31	81,60		31	52,50	
≥ 2500 grammes	39	63,90	7	18,40	0,0000	28	47,50	0,1020
	(n = 61)		(n = 38)			(n = 59)		

* Les différences sont calculées par rapport à la population du groupe I

### Morbi-mortalité maternelle

Aucun décès maternel n'a été déploré parmi les 162 accouchées des trois groupes. Concernant la morbidité post-partale, l'on a enregistré 6 cas de complications infectieuses (9,4%) parmi les accouchées du groupe I, il s'agit de 3 cas d'infections pariétales et 3 cas d'infections mixtes; 2 cas de complications traumatiques (3,4%) parmi les accouchées du groupe III (une déchirure cervicale hémorragique et un cas de déchirure vaginale). Aucune complication n'a été notée chez les accouchées du groupe II. L'analyse statistique ne montre pas de différence significative quant au risque global de complications entre les 3 groupes (p = 0,08 et 0,27) par contre le risque de complications infectieuses est statistiquement plus élevé chez les accouchées du groupe I (9,4%) comparativement à celles du groupe III (OR = ind.; p = 0,028) ou les groupes II et III réunis (OR = ind.; p = 0,0032). Le séjour hospitalier maternel moyen est de 12,97 jours (DS = 5,17) chez les accouchées par VH contre respectivement 2,54 jours (DS = 1,06) et 2,21 jours (DS = 1,60) pour les accouchées du groupe II et III et la différence est statistiquement significative entre le séjour hospitalier moyen du groupe I par rapport aux deux autres groupes d'accouchées (p = 0,0000) tel que le montre le Tableau 3.

**Tableau 3:** Morbidité maternelle

	N	%	p	OR ajusté	IC à 95%
**Hémorragies**					
Groupe I (n = 64)	0	0,00	-	1	-
Groupes II et III (n = 98)	1	0,01	1,0000	0,00	(0,00-26,84)
					
**Lésions des parties molles**					
Groupe I (n = 64)	0	0,00	-	1	-
Groupes II et III (n = 98)	1	0,01	1,0000	0,00	(0,00-26,84)
					
**Infections**					
Groupe I (n = 64)	6	9,40	0,0032	ind	-
Groupes II et III (n = 98)	0	0,00	-	1	-
					
**Séjour hospitalier anormal**					
Groupe I * (n = 62)	59	95,16	0,0000	0,01	(0,00-0,03)
Groupe II ** (n = 35)	5	14,29	0,72	0,63	(0,13-3,04)
Groupe III ** (n = 42)	4	9,52	-	1	-

* Le séjour hospitalier était considéré anormal lorsqu'il allait au-delà de neuf jours pour des raisons médicales évidentes

** Le séjour hospitalier était considéré anormal lorsqu'il allait au-delà de trois jours pour des raisons médicales évidentes

### Morbi-mortalité périnatale

#### Dépression néonatale

***A la fin de la première minute:*** le score d'APGAR a varié entre 1 et 9 chez les nouveau-nés du groupe I autour d'une moyenne de 6.04 (DS = 1,76) contre 1 et 9 et 2 et 10 dans les groupes II et III avec des moyennes respectives calculées à 6,6 (DS = 1,71) et 5,5 (DS = 2,08). La comparaison de ces moyennes ne donne pas de différence statistique significative (p = 0,07). De manière globale, 66% des nouveau-nés du groupe I et 60,7% du groupe III sont nés déprimés (score < 7) (p = 0,82) contre 31,40% dans le groupe II et la différence est statistiquement significative entre le groupe II par rapport aux deux autres (p = 0,0035).

***A la fin de la cinquième minute:*** le score a varié entre 2 et 10 dans le groupe I et le groupe III autour des moyennes respectives de 7,95 (DS = 1,89) et 7,07 (DS = 2,41) contre une variation de 4 à 10 avec une moyenne de 7,90 (DS = 1,42) dans le groupe II. La comparaison de ces trois moyennes ne montre pas de différence statistique significative (p = 0,14) de même qu'il n'en existe pas concernant la comparaison des risques de dépression néonatale (OR =1,75; IC: 0,41-7,86 et OR = 0,50; IC: 0,14-1,74).

***A la fin de la dixième minute:*** le score a varié entre 4 et 10 dans le groupe I, 5 et 10 dans le groupe II et 2 et 10 dans le groupe III avec des moyennes respectives de 8,67 (DS = 1,51), 8,56 (DS = 1,27) et 7.95 (DS = 2,43) pour lesquelles l'analyse statistique ne montre pas différence significative (p = 0,28) autant qu'il n'en existe entre les différents taux des nouveau-nés avec un score inférieur à 7 et représentant respectivement 8,82%, 6,70% et 17,39% (OR =1,37; IC: 0,17-12,69 et OR = 0,46; IC: 0,07-2,82). Les détails sur la dépression néonatale sont présentés dans le Tableau 4.

**Tableau 4:** Score d'APGAR

	Groupe I		Groupe II			Groupe III		
	n	%	n	%	p*	n	%	p*
**1**^**ère**^**minute**								
≤3	3	6,00	2	5,70	1,0000	5	17,85	0,1270
4-6	30	60,00	9	25,70	0,0037	12	42,85	0,2240
≥7	17	34,00	24	68,60	0,0035	11	39,30	0,8252
	(n = 50)		(n = 35)			(n = 28)	
**5** ^**ème**^ **minute**								
≤3	1	2,50	0	0,00	1,0000	4	14,80	0,1490
4-6	7	17,50	4	12,50	0,7442	5	18,50	1,0000
≥7	32	80,00	28	87,50	0,5958	18	66,70	0,3451
	(n = 40)		(n = 32)			(n = 27)	
**10** ^**ème**^ **minute**								
≤3	0	0,00	0	0,00	-	3	13,04	0,0600
4-6	3	8,82	2	6,70	1,0000	1	4,35	0,6406
≥7	31	91,18	28	93,30	1,0000	19	82,61	0,4230
	(n = 34)		(n = 30)			(n = 23)	

* Les différences sont calculées par rapport à la population du groupe I

### Complications néonatales: (Tableau 5)

33 nouveau-nés du groupe I (86,84%) n'ont connu aucune complication néonatale contre 25 (78,12%) et 15 (75%) dans les groupes II et III (p = 0,2782). L'anoxie néonatale est la complication la plus rencontrée et ce de manière presque égale dans les 3 groupes avec des taux respectifs de 13,16%, 15,63% et 15% alors que l'infection néonatale n'a été retrouvée que chez les nouveau-nés des groupes II et III avec des proportions voisines de 6,25% et 5% respectivement. Une élongation du plexus brachial a été enregistrée parmi les nouveau-nés du groupe III. Dans l'ensemble le risque de complications néonatales n'est pas statistiquement différent dans les 3 groupes (p > 0,05). Concernant l'issue néonatale, le taux de déperdition néonatale est de 12% chez les nouveau-nés du groupe I contre respectivement 22,9% et 11,1% dans les groupes II et III. La comparaison de ces différents taux ne donne pas de différence statistiquement significative (p = 0,31). Il en est de même du risque létal néonatal en rapport avec les 3 groupes étudiés. Le séjour hospitalier néonatal a varié entre 1 et 37 jours dans le groupe I avec une moyenne de 3,25 jours (DS = 5,31) contre 2 et 23 jours dans le groupe II et 1 et 8 jours dans le groupe III autour des moyennes respectives de 5,2 jours (DS = 4,74) et 2,6 jours (DS = 2,05). La comparaison des 3 moyennes ne montre pas de différence significative (p = 0,09) mais le risque d'un séjour hospitalier allongé (>3 jours) est statistiquement plus élevé concernant les nouveau-nés du groupe II comparé aux groupes I (OR = 7,24; IC = 1,92-28.91, p = 0,001) et III (OR= 0,20; IC= 0,04 - 0,84, p = 0,023).

**Tableau 5:** Morbi-mortalité néonatale

		n	%	p	OR ajusté	IC à 95%
**Traumatismes**						
	Groupe I (n = 38)	0	0,00	-	1	-
	Groupe II (n = 32)	0	0,00	-	-	-
	Groupe III (n = 20)	1	5,00	0,3448	0,00	(0,00-9,28)
**Anoxie néonatale**						
	Groupe I (n = 38)	5	13,16	-	1	-
	Groupe II (n = 32)	5	15,63	1,0000	0,82	(0,18-3,73)
	Groupe III (n = 20)	3	15,00	1,0000	0,86	(0,15-5,24)
**Infections néonatales**						
	Groupe I (n = 38)	0	0,00	-	1	-
	Groupe II (n = 32)	2	6,25	2,2053	0,00	(0,00-3,46)
	Groupe III (n = 20)	1	5,00	0,3448	0,00	(0,00-9,38)
**Décès néonatal**						
	Groupe I (n = 50)	6	12,00	1,0000	0,92	(0,16-4,69)
	Groupe II (n = 35)	8	22,90	0,1209	0,42	(0,00-2,06)
	Groupe III (n = 27)	3	11,10	-	1	-
Séjour hospitalier anormal*						
	Groupe I (n = 44)	5	11,36	-	1	-
	Groupe II (n = 27)	13	48,15	0,0014	7,24	(1,92-28,91)
	Groupe III (n = 26)	4	15,38	0,7177	1,42	(0,28-7,01)

* Le séjour hospitalier était considéré comme anormal lorsqu'il allait au-delà de trois jours pour des raisons médicales évidentes

## Discussion

L’étude relève une fréquence de 0,3% des parturitions en présentation transverse, ce qui représente un cas sur 333 accouchements. Cette fréquence est tout à fait voisine des taux rapportés dans la littérature variant entre un cas sur 200 à un sur 500 [1, 2, 4]. Il existe jusqu’à ce jour une réelle controverse parmi les auteurs quant au choix de la voie d'accouchement dans les parturitions en présentation transverse [8, 9, 13, 23–26], toutefois un consensus semble se dégager en faveur de l'opération césarienne considérée par beaucoup d'auteurs comme moins morbide vis-à-vis de la mère et du fœtus en comparaison avec la VB après manœuvre de version externe ou interne suivie de la grande extraction du siège, cette dernière modalité étant considérée par les défenseurs de la voie haute comme hautement morbide pour la mère (hémorragie, lésions vésicales, rupture utérine...) et le nouveau-né (dépression néonatale, traumatismes...) [10–12, 14]. Des 162 parturientes, 98 MVIGES ont été réalisées avec succès sur 103 tentatives soit un taux de réussite équivalent à 95,15%. Il y avait 39 VIGES réussies pour 41 tentatives (95,12%) sur J2 et 59 pour 62 (95,16%) sur contenu fœtal singleton. Il n'y a pas de différence significative lorsqu'on compare les taux d’échecs en rapport avec le contenu initial de l'utérus (deuxième jumeau ou singleton) (p = 1,0000).

Dans l'optique privilégiant la césarienne, les 98 VIGES ayant conduit à l'accouchement par VB représentent une diminution de 60,5% du taux attendu des césariennes dans cette série. L’étude révèle que les 3 groupes étudiés ne sont pas différents quant aux principales caractéristiques sociodémographiques (âge maternel et parité) et l'environnement obstétrical marqué par un taux élevé d’évacuations sanitaires (61,3% et 98,3%) et des membranes fœtales déjà rompues (95,2% et 96,6%) en ce qui concerne les groupes I et III. L’âge gestationnel montre un taux de grossesse à terme non différent statistiquement et que par ailleurs ces 2 groupes ne présentent pas de différences statistiques significatives quant au poids moyen de naissances des nouveau-nés (2780,16 grammes et 2380 grammes; p = 0,10) avec des proportions respectives de 63,93% et 47,46% de nouveau-nés d'au moins 2500 grammes. Enfin dans près de 96% des cas la VIGES a été réalisée sans narco-analgésie. Les similitudes présentées par les trois groupes concernant les caractéristiques sociodémographiques et l'environnement obstétrical garantissent que les différences susceptibles d'exister dans la morbidité périnatale et l'issue néonatale sont essentiellement justifiables par le mode d'accouchement choisi qui est dès lors la seule différence majeure entre ces trois groupes. Un taux de succès de plus de 95% dans les tentatives des VIGES chez des évacuées tardives (dilatation complète) avec ouverture de l’œuf totalisant plusieurs heures et sans narco-analgésie dans la plupart des cas, tous ces faits observés sont la preuve que la VIGES est une opération tout à fait faisable. L’âge maternel, la parité, l’âge gestationnel, l’état des membranes ne conditionneraient donc pas à eux seuls les chances de succès ou d’échec de la MVIGES. DUFOUR [27] et ANDRIAMADY [28] rapportent un taux d’échec variant entre 4 et 6% dans leurs tentatives de VMI et ce, sous anesthésie ou analgésie. Le taux élevé de succès observé dans cette étude serait tout simplement dû au strict respect des conditions de faisabilité (dilatation complète) et à la totale observance des contre-indications de la VB dans un contexte où le recours à la césarienne requiert une entière implication financière de la famille, la maternité ne disposant ni des trousses opératoires d'urgence ni des médicaments pour la gestion postopératoire. Les taux de complications rapportés dans les trois groupes ne sont pas statistiquement différents, toutefois l’étude révèle un risque infectieux plus élevé chez les accouchées par VH comparativement aux accouchées par VB (p = 0,0000). DUFOUR [27] dans une série des 35 VMI en France rapportait un taux de 8,5% d'hémorragies de la délivrance en 1996, ANDRIAMADY [28] au Madagascar relève dans une étude portant sur 177 VMI, 8% de ruptures utérines, 5% de déchirures cervicales, 73% d'hémorragies de la délivrance et 11% de décès maternels dus à l'hémorragie dans les 2/3 des cas et l'infection post opératoire pour les autres. La morbidité infectieuse chez les accouchées par voie haute est fort élevée et représente plus d'une opérée sur 10 dans notre étude. Elle est comparable à celle rapportée par d'autres études dans notre milieu comme ailleurs dans la plupart des pays pauvres et pourrait s'expliquer par les conditions obstétricales délétères dans un environnement sanitaire précaire [1, 2, 4]. Par contre, le taux bas de morbidité maternelle observé chez les accouchées après VIGES dans notre série, comparable aux taux occidentaux [6] mais contrastant ceux rapportés par ANDRIAMADY [28] se justifierait par les mêmes raisons qui ont été évoquées pour expliquer le haut taux de succès de VMI, à savoir: le strict respect des conditions de sa faisabilité et des contre-indications de la VB comme nous avons eu à le dire plus haut et permet d'affirmer que la MVIGES n'est pas si délétère pour la mère dans nos conditions de travail et que l'opération césarienne ne semble pas lui être supérieur comme en témoigne en plus de la moindre morbidité observée, la différence hautement significative du séjour hospitalier (13 jours contre 2 jours, p = 0,0000) et le coût financier y afférant.

L’étude révèle que la parturition sur présentation transverse est greffée d'un taux fort élevé de décès anténatals: 40 sur 162 (24,7%). Cette mortinatalité représente un cas sur 2 chez les parturientes du groupe III contre un sur 10 dans les groupes I et II (p = 0,0000); en clair, c'est dans les parturitions à contenu singleton que le taux des mortinaissances est extrêmement élevé. Ces résultats sont comparables à ceux rapportés par beaucoup d'auteurs africains [18, 19, 22, 28–31]. Dans la majorité des cas, il s'agit des parturientes évacuées tardivement des maternités périphériques, membranes longtemps ouvertes et après des manipulations irrationnelles et infructueuses par un personnel non qualifié. Enfin, comparés aux nouveau-nés du groupe I et du groupe III, les nouveau-nés J2 ont présenté un taux significativement plus faible de dépression néonatale à la première minute de naissance mais l’état de bien-être n'a pas montré de différence entre les trois groupes au delà de la 4ème minute signifiant que la différence observée à la naissance quant au taux de dépression néonatale n'est pas liée au mode d'accouchement mais à l'environnement obstétrical qui a entouré la parturition dans les 3 groupes, environnement réellement plus délétère dans les parturitions à grossesses monofœtales marquées par ailleurs par un taux élevé des évacuations tardives avec une part importante de mortalité anténatale témoin du climat parturitionnel [20, 21, 29, 32]. C'est d'autant plus vraisemblable que la césarienne n'a pas montré de supériorité concernant le risque de dépression néonatale face à la VB alors que ces bienfaits sont bien reconnus dans ses indications électives. A part un cas de complication traumatique enregistré parmi les nouveau-nés du groupe III, l'ensemble de la morbidité répertoriée dans les 3 groupes ne pourrait donc être imputable à la voie d'accouchement mais aux circonstances entourant la parturition, circonstances généralement communes et caractérisées par une carence dans la surveillance intrapartale, le sous-équipement, le manque de personnel qualifié sans oublier le rôle que joue encore l'ethnoculture dans nos sociétés pronatalistes qui sacralise la VB quel qu'en soit le prix, d'où un taux élevé des souffrances fœtales méconnues ou négligées donnant lieu à des nouveau-nés déprimés, fragilisés. Il s'agit donc d'un problème global et commun à la plupart des régions non développées tel que le rapportent plusieurs auteurs [19, 21, 29, 32].

S'agissant de l'issue néonatale, il a été déploré 17 décès néonatals sur 112 nouveau-nés pour lesquels l'information a été précisée, soit un taux de mortalité néonatale de 151,8% naissances. Le risque létal est évalué à 12,0% chez les nouveau-nés par VH contre 22,9% pour les deuxièmes jumeaux nés après VIGES (p = 0,3025) et 11,1% pour les singletons accouchés par MVIGES (p = 1,0000). Bien qu'il n'existe pas de différence statistiquement significative à la comparaison de ces différents taux, cliniquement parlant, la mortalité néonatale semble plus faible parmi les nouveau-nés par VH et les singletons nés après MVIGES, lesquelles modalités d'accouchement présentent elles, des taux de mortalité cliniquement et statistiquement identiques. Le fait que près de 57% des nouveau-nés J2 avaient un âge gestationnel inférieur à 38 SA et 82% d'entre-eux nés avec un poids inférieur à 2500 grammes peut expliquer la forte déperdition néonatale dans ce groupe dont la fragilité ne peut être efficacement assumée dans nos conditions de travail [32] par contre les taux similaires de mortalité néonatale observés chez les nouveau-nés par césarienne et ceux singletons nés par MVIGES démontrent que le risque létal en période néonatale n'est pas imputable à la voie de terminaison de l'accouchement mais à l'environnement obstétrical par ailleurs identique ayant prévalu dans les 2 groupes, environnement caractérisé par un taux élevé d’évacuations sanitaires (68% et 98,3%) et d'ouvertures des membranes fœtales (95,2% et 96,6%). D'ailleurs, le fait que le risque létal et le séjour hospitalier moyen chez les nouveau-nés par césarienne soient comparables à ceux des nouveau-nés par VB après manœuvres endo-utérines prouve d'une part que la césarienne n'a pas d'elle-même de pouvoir miraculeux, et d'autre part que la MVIGES n'est pas du tout aussi fœticide qu'on le prétend.

## Conclusion

La parturition en présentation transverse n'est pas un événement rare dans notre milieu, il est caractérisé par un environnement obstétrical précaire et la MVIGES pratiquée dans le strict respect des conditions de sa réalisation et des contre-indications de la VB, est une opération faisable, non délétère et non fœticide et qu'elle constitue de ce fait une alternative véritablement heureuse à la césarienne, celle-ci n'ayant pas montré de supériorité quant à la morbidité maternelle et l'issue néonatale dans nos conditions de travail. Elle devrait donc désormais faire partie de l'arsenal usuel de l'obstétricien œ'uvrant dans les régions à faible pénétration sanitaire.
